# Sclerosing epithelioid fibrosarcoma associated with *WRN* gene variant presenting as chronic dyspnea and pathologic cervical fracture: a case report and review of the literature

**DOI:** 10.1186/s13256-023-04249-6

**Published:** 2023-12-17

**Authors:** Alexander T. Phan, Henrik Ghantarchyan, Chayanne Khosravi, Bahareh Maknouni, Ankur Bhagat, Jeff Chen, Ahmad Ibrahim, Mufadda Hasan

**Affiliations:** 1 Department of Internal Medicine, Arrowhead Regional Medical Center, 400 N. Pepper Avenue, Colton, CA 92324 USA; 2 Department of Pulmonary and Critical Care Medicine, Arrowhead Regional Medical Center, Colton, CA 92324 USA; 3 Department of Pathology, Arrowhead Regional Medical Center, Colton, CA 92324 USA; 4 California University of Science and Medicine, Colton, CA 92324 USA

**Keywords:** Bronchoscopy, Thoracic oncology, Sclerosing epithelioid fibrosarcoma, Pathology, Pulmonology

## Abstract

**Background:**

Sclerosing epithelioid fibrosarcoma is an aggressive sarcoma subtype with poor prognosis and limited response to conventional chemotherapy regimens. Diagnosis can be difficult owing to its variable presentation, and cases of sclerosing epithelioid fibrosarcoma are rare. Sclerosing epithelioid fibrosarcoma typically affects middle-aged individuals, with studies inconsistently citing gender predominance. Sclerosing epithelioid fibrosarcoma typically arises from the bones and soft tissues and often has local recurrence after resection and late metastases. Immunohistochemical staining typically is positive for mucin-4. Werner syndrome is due to an autosomal recessive mutation in the *WRN* gene and predisposes patients to malignancy.

**Case presentation:**

A 37-year-old Caucasian female presented to the emergency department with 4 months of dyspnea and back pain. She had been treated for pneumonia but had persistent symptoms. A chest, abdomen, and pelvis computed tomography showed near-complete right upper lobe collapse and consolidation, mediastinal lymphadenopathy, lytic spinal lesions, and a single 15-mm hypodense liver nodule. The patient underwent a transthoracic right upper lobe biopsy, bronchoscopy, endobronchial ultrasound with transbronchial lymph node sampling, and bronchoalveolar lavage of the right upper lobe. The bronchoalveolar lavage cytology was positive for malignant cells compatible with poorly differentiated non-small cell carcinoma; however, the cell block materials were insufficient to run immunostains for further investigation of the bronchoalveolar lavage results. Consequently, the patient also underwent a liver biopsy of the liver nodule, which later confirmed a diagnosis of sclerosing epithelioid fibrosarcoma. Next-generation sequencing revealed a variant of unknown significance in the *WRN* gene. She was subsequently started on doxorubicin.

**Conclusion:**

Sclerosing epithelioid fibrosarcoma is a very rare entity, only cited approximately 100 times in literature to date. Physicians should be aware of this disease entity and consider it in their differential diagnosis. Though pulmonary involvement has been described in the context of sclerosing epithelioid fibrosarcoma, this malignancy may affect many organ systems, warranting extensive investigation. Through our diagnostic workup, we suggest a possible link between sclerosing epithelioid fibrosarcoma and the *WRN* gene. Further study is needed to advance our understanding of sclerosing epithelioid fibrosarcoma and its clinical associations as it is an exceedingly rare diagnosis.

## Background

Sclerosing epithelioid fibrosarcoma (SEF) was first described by Meis-Kindblom *et al.* in 1995 [1]. SEF is a rare and aggressive sarcoma that is characterized by epithelioid cells with clear or eosinophilic cytoplasm, in a densely sclerotic stroma [2–5]. SEF typically has a low-grade histological appearance; however, it is a very aggressive malignancy with frequent local recurrences [3]. Immunohistochemical studies typically demonstrate positive staining for mucin-4 (MUC4), and fluorescence in situ hybridization studies typically show *EWSR1* rearrangements [2–5]. The primary site of SEF origin is typically the lower extremities, and metastasis to the lungs and bones is common [3–5]. Patients are typically diagnosed in the fifth decade of life, with a median age of 45 years at diagnosis [4]. Management typically involves surgical resection if the tumor is amenable, chemotherapy, and radiotherapy, though no treatment modality has proven to be superior [3, 4]. Prognosis for SEF is typically poor, as patients commonly have local recurrence following resection and chemotherapy has not been optimized for this aggressive sarcoma subtype [5].

Werner syndrome (WS) is a rare autosomal recessive disease caused by a gene mutation in the *WRN* gene, and it is classified as a premature aging disorder, with an estimated prevalence of 1:380,000 to 1:1,000,000. Patients with WS may have manifestations including osteoporosis (found in 91% of affected individuals), type 2 diabetes mellitus (71%), neoplasms (44%), and atherosclerosis (30%) [6, 7]. Notably, neoplasms affect a large portion of patients with WS, and the literature has yet to cite an association between SEF and *WRN* gene mutations. As a genetic mutation can be identified after careful interpretation of the results, there exists an unclear interpretation of these results, described as a variant of unknown significance (VUS). We present a rare case of SEF in a patient with a genetic test yielding VUS of the *WRN* gene.

### Literature review

A literature review was conducted on the number of cases previously published regarding patients diagnosed with SEF and with a gene mutation or a variant of unknown significance (VUS) of the *WRN* gene. We performed a systematic review for eligible cases through a search on PubMed using the following terms “sclerosing epithelioid fibrosarcoma” and “WRN.” There were no reported cases in literature.

## Case presentation

**Fig. 1:**
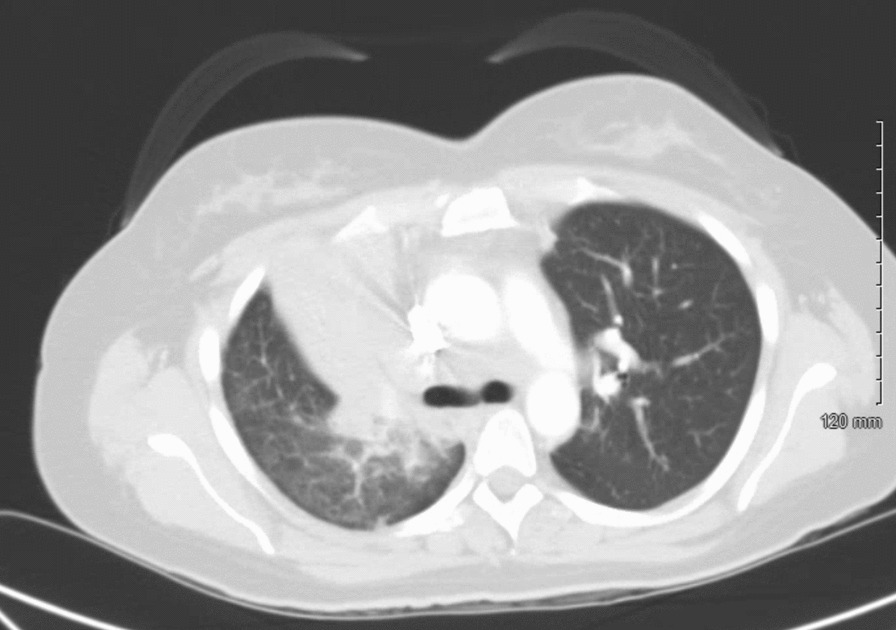
Axial section of a CT of the chest with intravenous contrast demonstrating right upper lobe consolidation with collapse

**Fig. 2:**
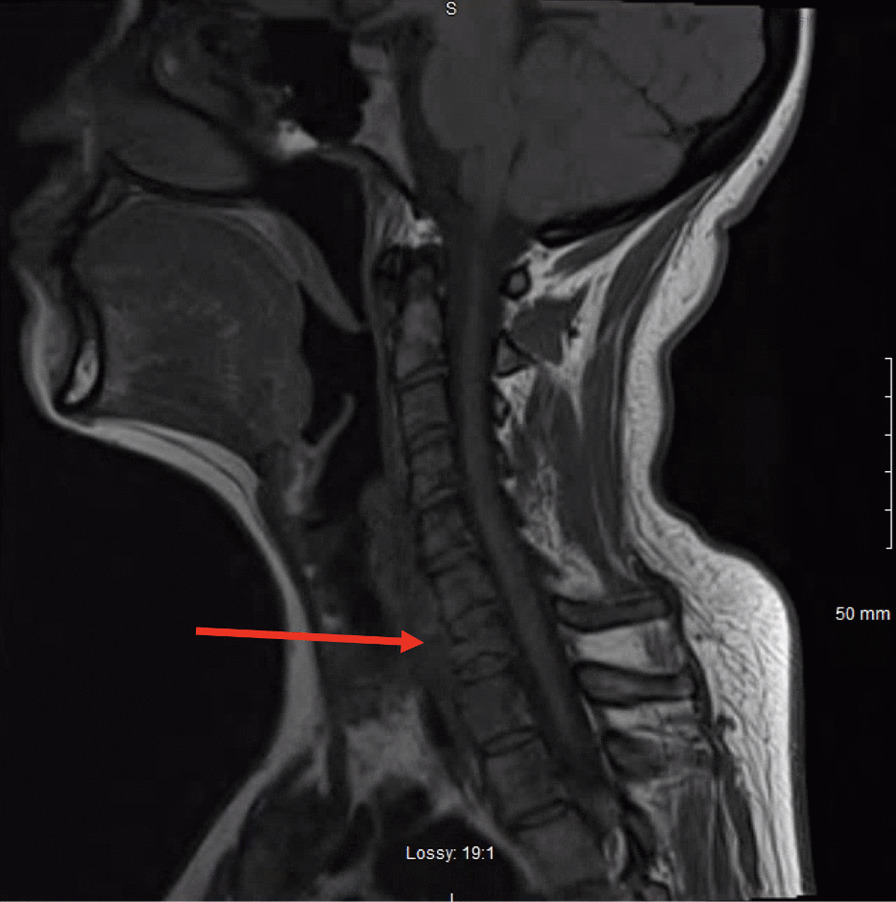
Sagittal section of a spine magnetic resonance imaging demonstrating a compression fracture at the level of C7 (red arrow)

**Fig. 3:**
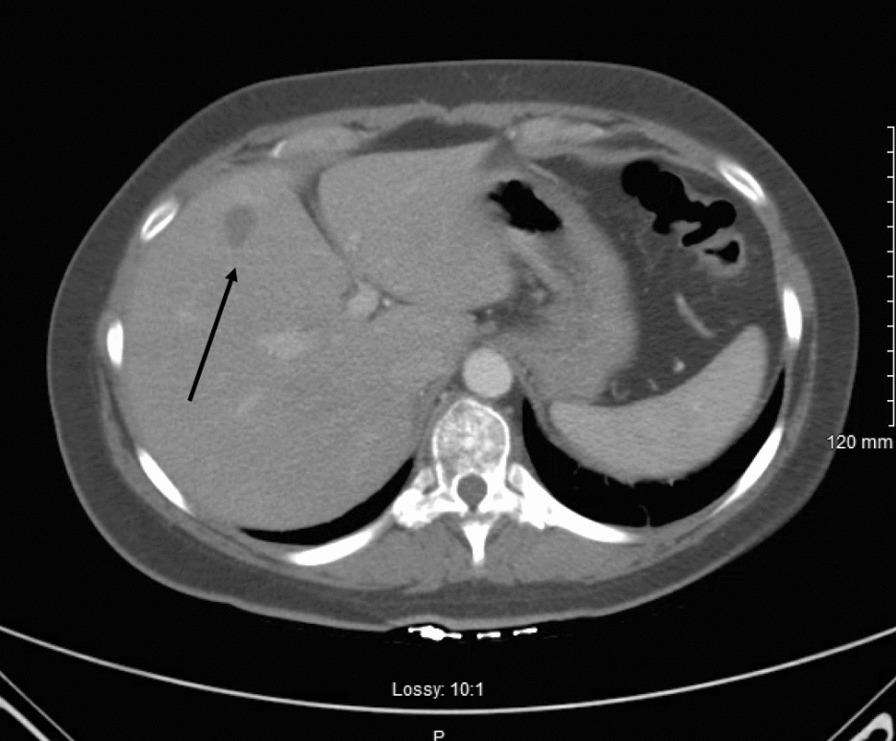
Axial section of a CT of the abdomen and pelvis with intravenous contrast revealing a 1.2-cm mass (arrow) in the right lobe of the liver

A 37-year-old Caucasian female nonsmoker with no known past medical history presented to our emergency department with 4 months of dyspnea and back pain. At her initial presentation 4 months prior to an outside facility, she underwent a chest C-ray and chest computed tomography (CT) scan, which showed a right upper lobe consolidation. She had been treated for and discharged with a diagnosis of pneumonia. Two months after her initial presentation, she underwent an outpatient bronchoscopy with bronchoalveolar lavage (BAL) and transbronchial right upper lobe biopsy owing to persistent symptoms and a concern for malignancy. Fungal studies and acid-fast bacilli fluorochrome smears were negative, and biopsy results were negative for malignant cells; the pathology results were consistent with chronic inflammation. Upon arrival to our emergency room, she again reported similar symptoms. Physical examination was notable for neck pain on palpation and tachypnea. A chest CT with intravenous (IV) contrast was performed, revealing near-complete right upper lobe collapse, right upper lobe consolidation, enlarged subcarinal lymph node, and enlarged station 6 lymph node (Fig. 1). Magnetic resonance imaging (MRI) of the spine was performed, demonstrating a C7 50% compression fracture (Fig. 2) and lytic lesions throughout the spinal vertebrae, concerning for metastatic disease. A CT of the abdomen and pelvis was also performed, revealing a single 15-mm hypodense nodule on the liver (Fig. 3).

The patient subsequently underwent a transthoracic right upper lobe biopsy, which showed dense fibrous pleural bits, samples of alveolar parenchyma showing interstitial fibrosis, and a patchy dense lymphocytic infiltrate; no malignant cells were seen on histopathological examination. Next, the patient underwent bronchoscopy and endobronchial ultrasound with transbronchial lymph node sampling, as well as brushing and BAL of the right upper lobe. Most studies were unremarkable except the BAL cytology exhibit, which was positive for poorly differentiated neoplastic cells, raising the possibility of a poorly differentiated carcinoma; however, the material on the cell block was insufficient to run immunostains. As the concern for metastatic disease remained high on our differential diagnosis, we pursued a liver biopsy to better characterize the malignancy. The patient underwent a fluoroscopy-guided liver biopsy of the aforementioned liver nodule, which demonstrated a malignant undifferentiated neoplasm with epithelioid morphology and weak neuroendocrine differentiation.

**Fig. 4:**
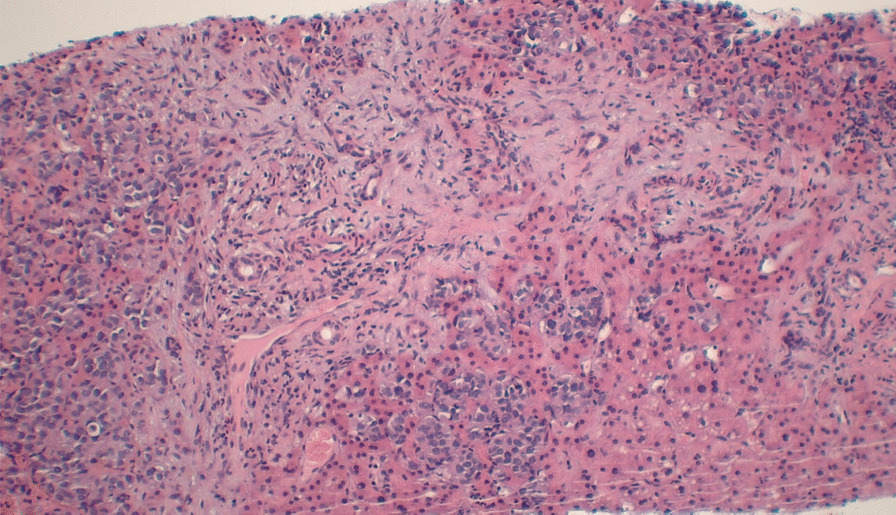
Neoplastic cells infiltrating liver tissue forming nests of epithelioid tumor cells (hematoxylin and eosin, 10×)

**Fig. 5:**
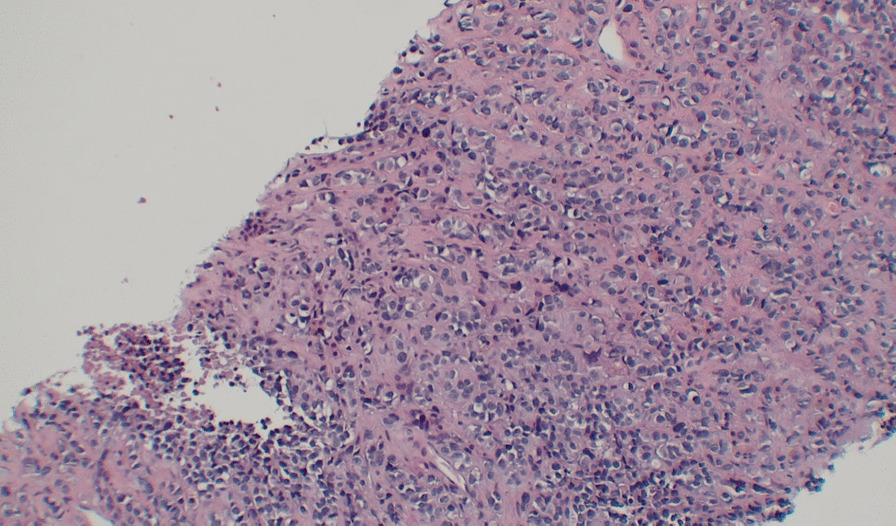
Characteristic feature of sclerosing epithelioid fibrosarcoma demonstrating a prominent hyalinized sclerotic collagenous stroma within which there is a relatively bland arrangement of monomorphic epithelioid cells in cords and nests (hematoxylin and eosin, 10×)

**Fig. 6:**
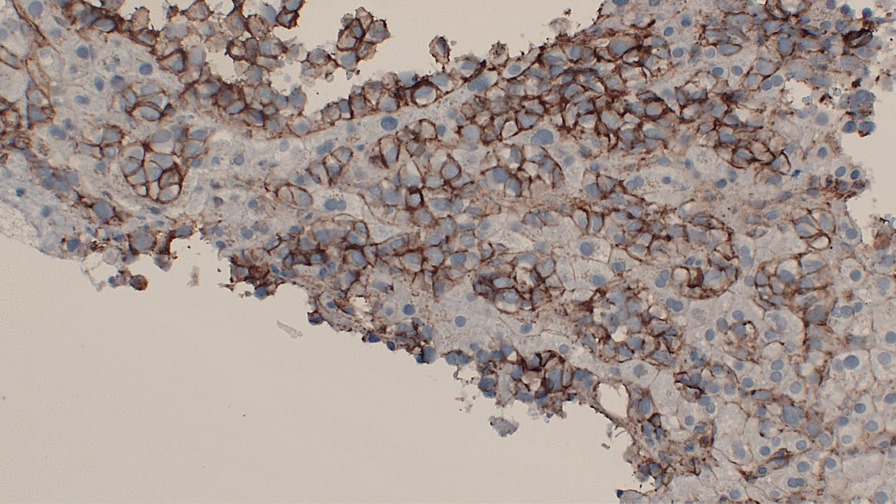
CD-99 membranous pattern of immunostaining (immunohistochemistry, 10×)

**Fig. 7:**
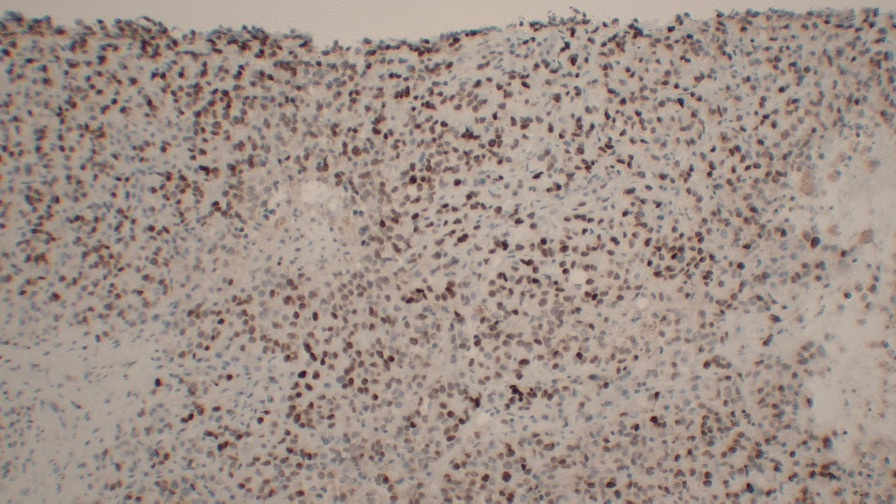
GATA-3-positive immunostaining (immunohistochemistry, 10×)

**Fig. 8:**
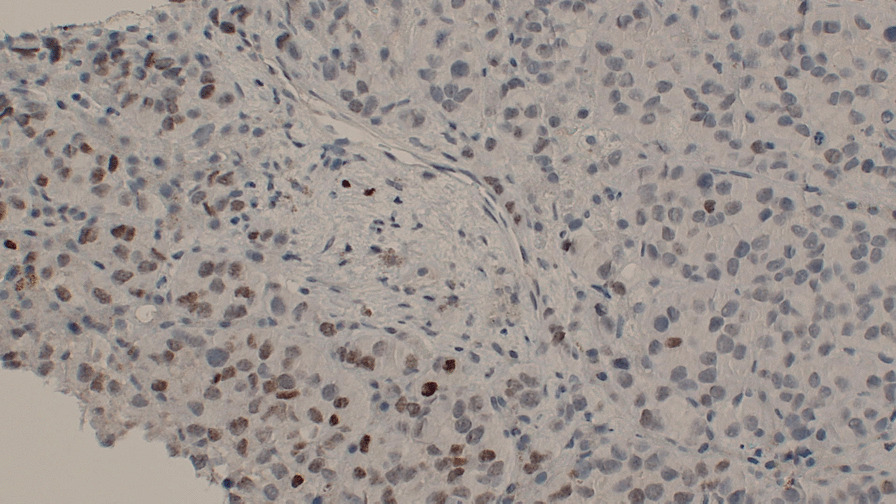
PAX-8 weakly positive immunostaining in a subset of neoplastic cells (immunohistochemistry, 10×)

The cells formed nests/cords in a background of hyalinized sclerotic stroma, frequently demonstrating eosinophilic cytoplasm and round-to-ovoid nuclei (Figs. 4 and 5). These ancillary studies were insufficient to rule out epithelioid synovial sarcoma, breast malignancy, renal malignancy, thyroid malignancy, gynecologic primary tumors, and epithelioid osteosarcoma. The neoplastic cells were weakly reactive for synaptophysin, and CD99 staining showed a membranous pattern (Fig. 6). In a subset, GATA-3 was positive and PAX-8 was weakly positive (Figs. 7 and 8). However, the neoplastic cells were nonreactive to ER (breast marker) and SATB2 (lower gastrointestinal and osteosarcoma marker). Moreover, the tumor cells were negative for Oscar pankeratin, CK7, CK20, Moc-31, TTF-1, WT-1, HMB45, Melan-A, SOX-10, S100, Inhibin, SMA, MyoD1, CD117, CD45, CD30, and CD34. Thus, the liver tissue was sent for a second opinion for evaluation by a pathologist with expertise in gastrointestinal, liver, soft tissue, and cardiothoracic pathology in a state-of-the-art center at the national and international levels. They found that a specialized panel of immunohistochemical stains revealed that the tumor cells were reactive to MUC4 expression. BRG1 and INI1 immunostains revealed preserved nuclear staining; however, claudin-4, ERG, CD31, CAMTA1, P40, ETV-4, and NUT stains were negative. Subsequently, a diagnosis of sclerosing epithelioid fibrosarcoma was concluded.

Based on our diagnostic workup, the patient was promptly started on doxorubicin therapy. The patient ultimately joined a genetic research protocol at a major cancer research center. Next-generation sequencing (Tempus genetic testing) was completed to evaluate for any variant known to increase her risk for cancer. There were no genes identified that would increase her risk for cancer. However, there was an identified variant of unknown significance of the *WRN* gene, an autosomal recessive gene associated with Werner syndrome (WS). She has not had any clear manifestations of WS other than malignancy and has yet to be assessed for osteoporosis. At 3-month follow-up, she is in the early stages of her treatment course and has tolerated her regimen without issues.

## Discussion

Sclerosing epithelioid fibrosarcoma (SEF) is a very rare disease entity, cited only approximately 100 times in literature to date [3]. Low-grade fibromyxoid sarcoma (LGFMS) shares morphological and molecular features with SEF, which has raised debate over distinguishing characteristics between the two neoplasms [2–5]. Though similarities exist, the clinical history, clinical course, and histopathology of the two disease entities help differentiate them from one another. First, SEF tends to affect individuals slightly later than LGFMS, with median diagnosis age of 45 years for SEF and 29 years for LGFMS. Second, SEF also tends to involve periosteal and osseous sites, while LGFMS typically does not. Third, SEF has a much more aggressive clinical course, evidenced by earlier and more diffuse metastasis [4]. Specifically, Doyle *et al.* demonstrated that mucin-4 (MUC4) has diffuse cytoplasmic staining in 78% of SEF cases and *EWSR1*–*CREB3L1* fusion gene rearrangement in 38% of cases, suggesting that these markers may be relatively sensitive and specific for SEF [2]. Fourth, though both SEF and LGFMS stain positive for mucin-4 (MUC4), SEF tends to harbor *EWSR1* rearrangements [2–5, 8]. Finally, the cytomorphology for SEF typically demonstrates nests and cords of epithelioid cells with clear or eosinophilic cytoplasm within a sclerotic stroma, while LGFMS demonstrates bland spindle cells arranged in a whorled pattern [8].

In reviewing the literature, the most common primary site for SEF is the lower extremities, with common complaints including thigh pain [3–5]. Our patient’s case is unique in that her primary complaint was chronic dyspnea and neck pain. Though the primary site of her SEF is unclear, three potential sites include the spine, lung, and liver. The spine is unlikely to be a primary site in this case because there were diffuse lytic and sclerotic lesions noted in the cervical, thoracic, and lumbar vertebrae, suggesting metastatic disease. The lung was our next consideration; however, as several studies have noted, the lungs are the most common site for SEF metastasis, and only one case by Leisibach *et al.* has ever reported a primary lung SEF, which was also in the setting of Lynch syndrome [3–5, 9]. These considerations make a primary lung SEF less likely in our case. Chew *et al.* and Warmke *et al.* identified a total of five intraabdominal cases of primary SEF, and one case report has described a primary liver SEF [3, 4, 10]. The patient in our case had a solitary liver lesion whose cytomorphology and immunohistochemical staining was consistent with SEF, and taking into consideration the typical clinical course of SEF and the case of our patient, the most likely primary site is the liver.

The treatment of SEF may involve surgical resection of localized disease, chemotherapy, and radiotherapy; however, local recurrence following resection is common. Chemotherapeutic agents have limited benefit in SEF, with common regimens including doxorubicin, ifosfamide, gemcitabine, and docetaxel [3–5]. Chew *et al.* describe seven patients with metastatic SEF who received chemotherapy, where three patients received doxorubicin monotherapy, three other patients received a combination of doxorubicin and ifosfamide, and one patient received a combination of gemcitabine and docetaxel. The patients who received doxorubicin monotherapy had limited stable disease (range 1.2–7 months), while the other patients had either a partial response or no response at all [3]. These results highlight the dearth of data available regarding SEF treatment, the poor prognosis associated with SEF, and the limited ability clinicians have in the management of SEF.

The patient in our case also had a variant of the *WRN* gene, which encodes a helicase that plays a role in chromosome stability [6]. Werner syndrome is characterized by an autosomal recessive mutation in the *WRN* gene, and one of the major disease manifestations is cancer predisposition [6, 7]. Though our patient does not have a definitive diagnosis of WS, a variant of unknown significance in the *WRN* gene is concerning, as one of the most common causes of death in patients with WS is malignancy. Interestingly, Oshima *et al.* note that, in a meta-analysis of WS patients with cancer, there was a disproportionately higher incidence of sarcomas [7]. This may provide a link to the association between SEF and *WRN* gene variants; however, larger studies are needed to properly assess this association.

The primary limitation in our case is that we were unable to definitively identify the primary site of tumor origin. We decided to not pursue a surgical biopsy of the lung, as this would not have changed our management, nor would it have provided further diagnostic yield. Though this limitation exists, our suspicion is that the liver is the primary site of tumor origin based upon our previous discussion. Regarding treatment, there are no clear guidelines regarding the best therapeutic modalities; however, our patient is receiving doxorubicin infusions and has not had further disease manifestations. The strength of this case report lies in its novelty, namely SEF associated with a *WRN* variant. To our knowledge, no other case study has ever reported this association, and we hope to contribute to the current body of limited literature regarding SEF. Future studies should involve larger randomized clinical trials to better define treatment modalities for SEF and study the potential associations between *WRN* mutations and SEF. At this time, SEF still remains a rare diagnosis, and awareness of SEF is essential for clinicians and pathologists, as this malignancy has many histopathologic mimics and has a very aggressive clinical course.

## Conclusion

Through this case report, we hope to raise awareness of this rare sarcoma, its histopathological characteristics, and the limited therapeutic options available. We would also like to highlight a possible association between *WRN* gene variants and sclerosing epithelioid fibrosarcoma. Physicians should include SEF in the differential diagnosis when evaluating patients with a possibility of malignancy, as SEF portends a poor prognosis. Future studies may assess the associations between *WRN* variants and SEF, and also determine more optimal therapeutic regimens for SEF.

## Data Availability

All data in our report were obtained from the patient’s hospitalization. Any inquiries regarding supporting data availability of this study should be directed to the corresponding author.

## Abbreviations

SEF: Sclerosing epithelioid fibrosarcoma
MUC4: Mucin-4
CT: Computed tomography
BAL: Bronchoalveolar lavage
VUS: Variant of unknown significance
IV: Intravenous
mm: Millimeter
cm: Centimeter
WS: Werner Syndrome
LGFMS: Low-grade fibromyxoid sarcoma
GATA-3: Globin transcription factor 3
PAX-8: Paired-box gene 8
ER: Estrogen receptor
SATB2: Special AT-rich sequence binding protein 2
CK: Cytokeratin
Moc-31: Monoclonal antibody 31
TTF-1: Thyroid transcription factor 1
WT-1: Wilms tumor 1
HMB45: Human melanoma block 45
SOX-10: Sex-determining region Y-related high mobility group-box 10
SMA: Smooth muscle actin
MyoD1: Myogenic differentiation 1
CD: Cluster of differentiation
